# Effect of Recrystallization Behavior of AZ31 Magnesium Alloy on Damping Capacity

**DOI:** 10.3390/ma16041399

**Published:** 2023-02-07

**Authors:** Kibeom Kim, Yebin Ji, Kwonhoo Kim, Minsoo Park

**Affiliations:** 1 Department of Marine Design Convergence Engineering, Pukyong National University, 45 Yongso-ro, Nam-gu, Busan 48513, Republic of Korea; 2 Department of Metallurgical Engineering, Pukyong National University, 45 Yongso-ro, Nam-gu, Busan 48513, Republic of Korea; 3 Institute of Multidisciplinary Research for Advanced Materials (IMRAM), Tohoku University, Katahira 2-1-1, Sendai 980-8577, Japan

**Keywords:** AZ31 magnesium alloy, hot-rolling, annealing, static recrystallization, tensile twinning, fexture, damping capacity

## Abstract

For a wide industrial application of magnesium alloys, a method for imparting high damping properties while maintaining mechanical properties is required. Controlling the crystallographic texture seems to be useful, because dislocations are known to have a significant influence on the damping characteristics of magnesium alloys. In addition, textures are affected by the microstructure and texture variation when the deformation or annealing is applied. However, there were less reports about their effect on damping capacity. Therefore, the effect of twinning and annealing, which can affect the recrystallization, were investigated in this study. An AZ31 alloy was hot rolled at 673 K with a reduction ratio of 10% and 50%, and then annealed at 673 K and 723 K for 0.5, 1, 2, and 3 h, respectively. SEM-EBSD was used to examine the microstructure and texture. In addition, each specimen’s hardness and internal friction were contemporarily measured. As a result, hot rolling produced tensile twins and their fraction increased with internal friction when the reduction ratio increased. Due to annealing, a discontinuous type of static recrystallization occurred within the twinning grains, and was highly activated along with the increasing annealing temperature and the fraction of twinning. In the samples annealed at 723 K, the internal friction continuously increased over the annealing time, whereas in the samples annealed at 673 K, the decrease in dislocation density was delayed while the internal friction showed a relatively low value.

## 1. Introduction

Magnesium alloys are well known for their high vibration-damping capacity compared to other alloys. They have been highly regarded as materials that can protect products from external vibrations and reduce noise in the fields of electronic devices and transportation [1,2,3]. However, commercial magnesium materials are generally alloyed with various additive elements, such as Al or Ca, to improve their strength and mechanical properties. Although this can improve the mechanical properties, it lowers the damping ability [4,5]. Therefore, it is necessary to study ways to improve damping ability while maintaining high mechanical properties for industrial application. Granato and Lücke proposed a damping mechanism model in magnesium alloys [6]. Furthermore, the degree of damping capacity in magnesium alloys is determined by dislocation-type mechanism, which is a static hysteresis mechanism. Additionally, various types of lattice defects controlling the movement of dislocation contribute to the damping characteristics. Because the grain or phase boundaries are mostly affected on the damping properties of magnesium alloys, many studies have been conducted to enhance the damping property in the cast material by controlling the shape or distribution of the second-phase components [7,8,9]. However, it seems that the deformation will be more sensitive than casting, because the damping is a property affected by the dislocation densities inside the material.

Owing to a low number of active slip systems of magnesium alloys in most temperature ranges, the activation of twinning is essential during the deformation process. Through the generation of twinning, additional slip systems required for deformation are supplied. [10] In addition, the occurrence of such a large fraction of twins serves as a place where static recrystallization occurs subsequently with recovery in the annealing process. [11] This means that an increase in the twinning fraction means the formation of a higher dislocation density in the microstructure, and recrystallization can appear more actively. Therefore, the generation of dislocations or twinnings by deformation, and the recovery and recrystallization process during the annealing process appeared to affect the increase or decrease in dislocation density occurring in the microstructure of magnesium alloy. In addition, the development of texture orientation in magnesium alloy is known as a factor that has a great influence on plasticity anisotropy or workability compared to other alloys [12,13]. One of authors conducted research using magnesium alloys with different Al contents in alloy, such as AZ series magnesium alloys [14,15]. Through these results, the formation of textures can be controlled by the variation in deformation factors such as solute atoms, deformation rate, and temperature. Considering that these factors are influenced by the activity of dislocations, they can also affect the damping performance. Furthermore, authors have investigated the distribution of dislocation density using the Kernel average misorientation map [16]. Through them, the annealing process or deformation in high temperature environment made the dislocation density to be changed. However, it seems that reported studies about the relationship between the texture and damping capacity are still insufficient. Moreover, because twinning and recrystallization (REX) is an important mechanism to accompany the deformation in Mg alloys and the dislocation densities would be varied by the development of microstructure, their effect should be investigated prior to investigate. In particular, because the changes in these dislocation densities can also contribute to the mechanical properties of alloys such as a strength or hardness, it seemed necessary to understand the process effects on damping properties by measuring them.

Therefore, this study was conducted to investigate the effect of twinning and REX on damping ability in magnesium alloy, the observation of damping capacity after hot rolling and annealing to AZ31 (Mg-3wt% Al-1wt% Zn) alloy.

## 2. Materials and Methods

### 2.1. Experimental Procedure

Commercial cast AZ31 alloy was used as the material, and they were obtained from the Suzhou Chuanmao Metal Material.co. To determine the chemical composition, 5 different specimens were cut from the ingots in to 10 × 10 × 4 mm sizes and X-ray fluorescence ((XRF) XRF-1800, SHIMADZU, Tokyo, Japan) was obtained. Their chemical composition is shown in Table 1.

Firstly, the alloy was machined into a plate material with a size of 80 mm × 60 mm × 40 mm (length × width × thickness). They were rolled at 673 K with rolling reduction ratio of approximately 10 and 50%, respectively. To avoid the cracking and fracture as much as possible, the hot rolling process was performed by repeating the multi-pass process after setting the reduction ratio as approximately 2 to 3% per pass. Between each pass, the specimens were reannealed at 673 K for 600 s in the electric furnace.

**Figure 1:**
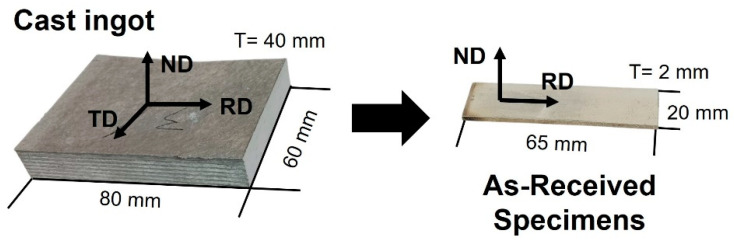
Images of the specimen of cast ingot and the as-received specimen.

After then, the specimens were cut from the rolled plate to have dimensions of 65 mm × 20 mm × 2 mm using microtom cutter (Struers, Copenhagen, Denmark) as shown in Figure 1. The perpendicular direction of the wide plane in the specimen was set to be the same as that in the normal direction (ND), and parallel to the specimen length direction was to be the same with rolling direction (RD). Specimens were annealed at the temperature 623 K and 723 K and the time for 0.5, 1, 2 and 3 h. After the annealing time passed, specimens were quenched in the water to prevent the variation in microstructure.

The annealed specimens were mechanically grinded using sandpaper and polished using OP-S abrasive with SiO_2_ particles to observe the microstructure and texture. Microstructural observations were performed using an optical microscope ((OM) GX51, Olympus, Tokyo, Japan) and a scanning electron microscope ((SEM) GX51, Olympus, Tokyo, Japan).

The polished specimens were etched in a picric acid solution (5 mL 70% Acetic acid + 45 mL 94% ethanol + 3 g picric acid) for 2 to 30 s, and then washed with ethanol, for the observation of microstructure using OM. In addition, prior to using the electron microscope, electropolishing was performed to polished specimen, using an electropolishing machine (Lectropol-5, Struers, Copenhagen, Denmark), with a current density of 0.18 to 0.22 A/cm^2^ implied to the specimen in 3% perchloric acid solution (15 mL HClO_4_ + 475 mL 99.99% Ethanol). More detailed methods are explained in ref. [16].

Hardness was measured to investigate the fraction of REX according to the reduction ratio and annealing conditions using, a Vickers hardness tester (Wolfert Wilson, 402MVD/SVD, BUEHLER, Lake Bluff, IL, USA). A diamond indenter was pressed on the surface of specimens for 10 s with a load of 0.3 N to leave an indentation. A CCD camera attached to the equipment was used to measure the pressed area.

**Figure 2:**
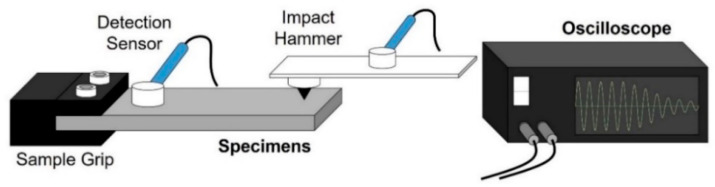
Schematic illustration of equipment for the observation of damping capacity.

In addition, an internal friction measuring device was used to measure the vibration damping ability, as shown in Figure 2. After one end of the specimen was fixed, the other end was vibrated using a hammer. Furthermore, the free decay waveforms decayed until they came to a complete stop, which was then measured as data using an oscilloscope. The analysis was performed by measuring the logarithmic decay rate as the waveform decayed.

### 2.2. Fractional Softening

Su et al. performed a hardness test to measure the magnitude of the REX of AZ31, which is the same alloy used in this study. Similarly, the Equation (1) was applied to quantify the degree of softening by REX [11].

(1) $$X_{H}=\frac{H_{0}-H_{annealing}}{H_{0}-H_{fullrex}}$$

Here, X_H_ means the fraction of softening, *H*_0_ is the hardness after rolling, *H_annealing_* is the hardness after heat treatment and *H_fullrex_* is the hardness after the REX was totally completed.

### 2.3. Internal Friction Coefficient

The logarithmic decay rate (δ, logarithmic decrement) is a factor that indicates the degree to which the free vibration for the next amplitude decreases when the specimen is in free vibration without external stress. This is represented by Equation (2) [2].

(2) $$\Delta =\frac{1}{n}\ln\left(\frac{A_{0}}{A_{n}}\right)$$

where *n* is the wave number, *A*_0_ is the initial wavelength amplitude, and *A_n_* is the amplitude of the *n*th wavelength. In addition, there is an internal friction coefficient (Q^−1^) as an index indicating the degree of actual vibration damping characteristics, and the logarithmic damping rate of free damping has a relationship as shown in Equation (3) below.

(3) $$Q{-}^{1}=\frac{\delta }{\pi }$$

## 3. Results

### 3.1. Microstructure Development

**Figure 3:**
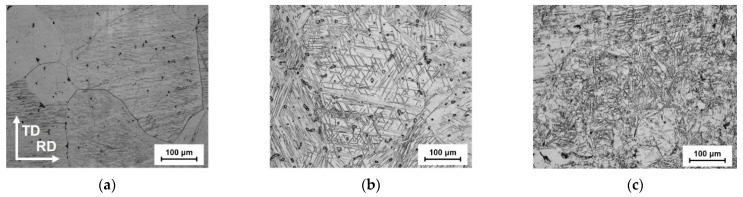
Microstructures of AZ31 alloy. Each figure shows the condition under (**a**) as-received and after rolling with the reduction rate of (**b**) 10% and (**c**) 50%.

Figure 3 shows the microstructure of cast ingots and that of hot rolling. Coarse grains with a maximum size of approximately 300 μm were observed as shown in Figure 3a. Twinning occurred in the microstructure after hot-rolling at two different reduction ratios of 10% and 50%. Moreover, the increment of rolling reduction formed more twinning.

**Figure 4:**
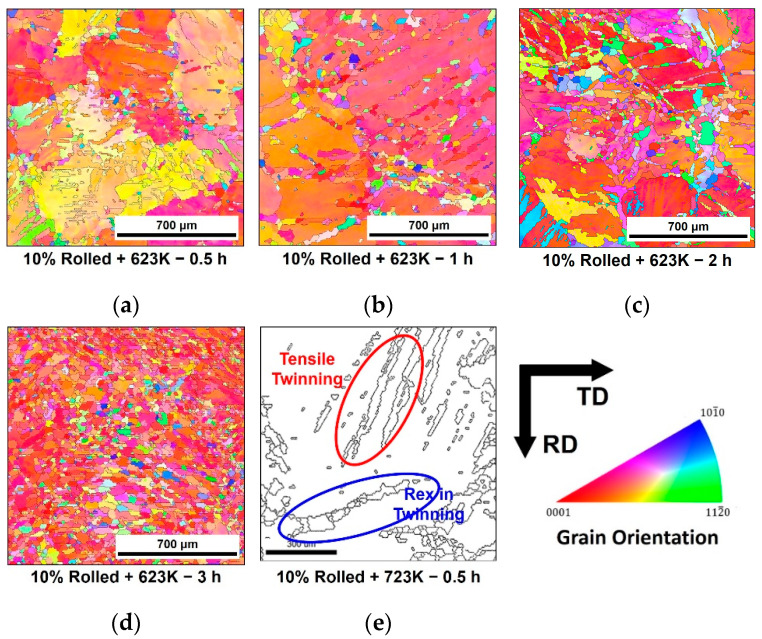
Microstructures of the specimens after rolling with the 10% reduction and annealing at 623 K for (**a**) 0.5, (**b**) 1, (**c**) 2 and (**d**) 3 h and (**e**) at 723 K for 0.5 h.

Figure 4 shows the microstructure observed after isothermal annealing at 623 K for a 10% reduction of rolled AZ31 specimen. Orientations of each grain were colored according to the standard triangle show in right side. Each image below shows the time conditions maintained during annealing. Fine and equiaxial grains were formed in the twinning grains during the initial stage of annealing process. As time progresses, twinning fades, and the fraction of these grains increase. Figure 4e shows an enlarged view of one of the microstructure. Fine grains were formed in twinning. Therefore, it could be confirmed that REX by annealing was occurred by annealing. In addition, after annealing under all conditions until the final stage, the twin structures generated by rolling were dissipated and completely replaced by equiaxial crystal grains.

**Figure 5:**
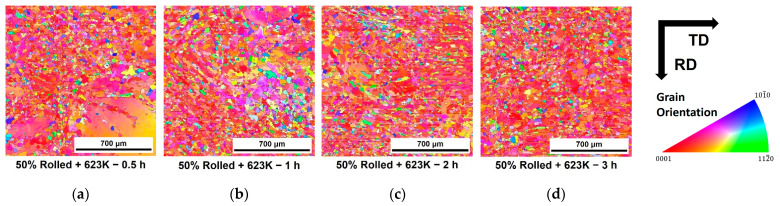
Microstructures of the specimens after rolling with the 50% reduction and annealing at 623 K for (**a**) 0.5, (**b**) 1, (**c**) 2 and (**d**) 3 h.

Figure 5 shows a specimen of AZ31 alloy rolled with a reduction of 50% in the same way of Figure 4. The microstructure behaviors during annealing and after the final condition were almost similar as that observed previously. However, the twinning disappeared more rapidly after 30 min, even though the fraction of twinning before annealing was higher in 50% than the 10%.

**Figure 6:**
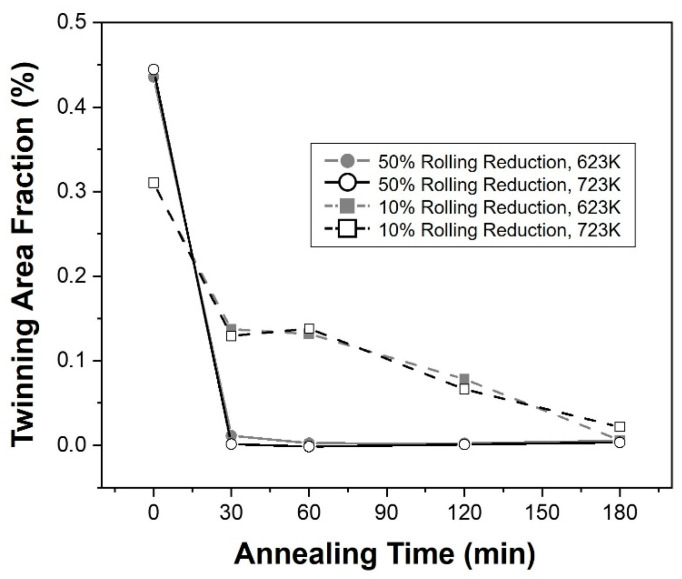
Graph showing the relationship between annealing time and twinning fraction in the result of microstructure.

Figure 6 shows the area fraction of twins observed in the microstructure measured under each condition according to the annealing time. In all conditions, as the annealing time increased, the twin fraction decreased. Although the twin fraction at a reduction of 50% was higher than that a 10% reduction before annealing, it rapidly decreased at the initial stage of annealing regardless of temperature, whereas a reduction of 10% showed a relatively gradual reduction.

**Figure 7:**
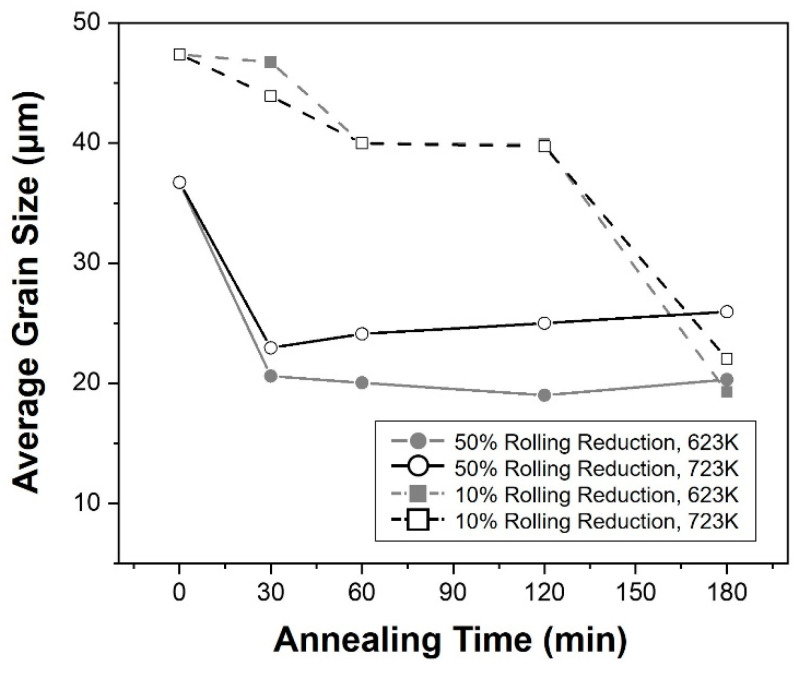
Graph showing the relationship between annealing time and average grain size after rolling with the reduction of 10% and 50%, respectively.

Figure 7 shows the relationship between the average grain size and the annealing time after rolling under two different conditions. As the reduction ratio increased for rolling, more twins were generated and a finer grain size appeared. When the specimens were rolled in 50%, the size of the crystal grains decreased dynamically in the initial stage, especially after the 0.5 h. However, their scale was remained without any significant variation until the final stage of annealing. On the contrary, the grain sizes after 10% rolling decreased slightly in the initial stage of annealing and remained until 2 h. After then, their grain sizes were decreased significantly. After the true strain-1.0, the grain sizes observed in each specimen became almost similar to each other, although their values were different before then. Following this point, the specimens under all conditions had similar final particle sizes and remained unchanged. Therefore, the specimens rolled in 10% reduction were fully recrystallized after 2 h, while those rolled in 50% reduction were after 0.5 h, respectively. The decision of H_fullrex_ value in Section 3.3 was used as the value of these points. Moreover, it was considered that the increase in reduction ratio made REX more active, supplying a driving force by enhancing the twinning generation.

### 3.2. Texture Development

**Figure 8:**
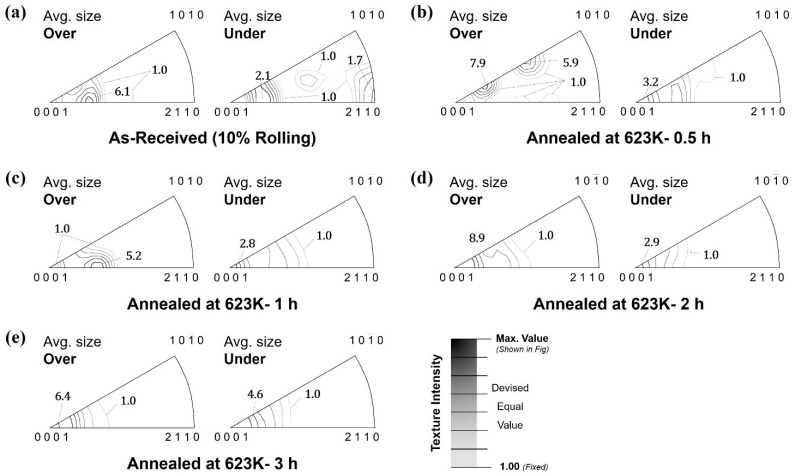
IPFs of the specimen (**a**) rolled with 10% reduction and after annealing at 623 K for (**b**) 0.5, (**c**) 1, (**d**) 2, and (**e**) 3 h.

Figure 8 shows the texture change in inverse pole figure (IPF) before and after annealing of the specimen rolled at 623 K with 10%. A mean axis density was used as a unit intensity. In addition, the texture intensities were shown in seven lines having values which are equally devised of values within the range from unit density to maximum density as shown in scale. The textures were separated into two different grain size ranges, based on the average grain size shown in Figure 7. Those larger than the average are shown on the left in each IPF set, and smaller ones are shown on the right.

As shown in Figure 4 and Figure 5, twinnings are formed by the hot-rolling and their grains were becoming smaller than their mother grains. After the annealing, the recrystallized grains were formed from the twinning grains. Accordingly, the grains which were obtained from the twinning and recrystallized had lower sizes than the average grain sizes. This made the texture on the right side of Figure 8a to have [11$\bar{2}$0] orientation in the initial state with the (0001) basal pole in same time. However, as shown in Figure 8b–e, this tilted orientation gradually disappeared as the annealing time increased, while the basal fiber structure gradually developed.

On the contrary, the grains larger than the average size are observed by the one before rolling or after growth stage. In these cases, the texture orientation was tilted from the basal pole toward the [11$\bar{2}$0] orientation by approximately 20~30°. This texture component has been reported in ref [17]. Similarly, to the lower sizes, their orientations were varied toward the basal pole after the annealing.

Through the annealing process, the texture orientation was varied to having basal pole, regardless of the grain sizes. Moreover, this behavior was more highly observed when the rolling reduction and the fraction of twinning is increased. The rapid variation in texture orientation seemed to occur due to the twinning and their recrystallization. These will be discussed in the later section.

### 3.3. Damping Capacity

**Figure 9:**
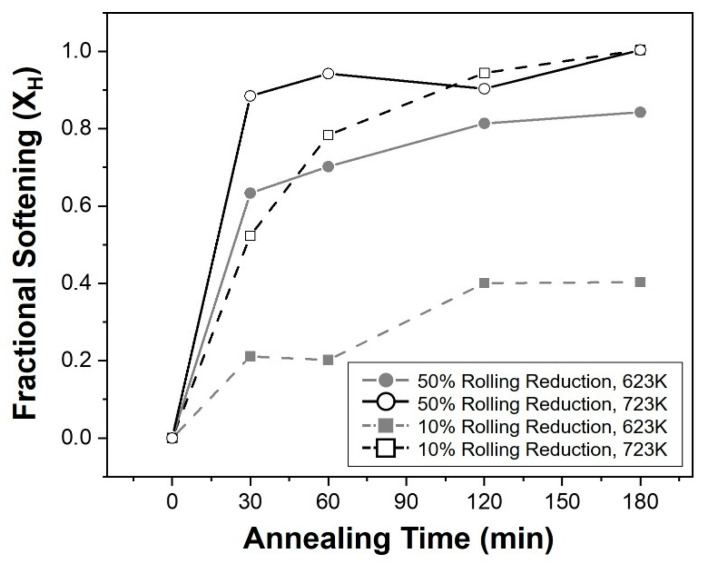
Graph showing the relationship between the fractional softening and annealing condition.

Figure 9 shows a graph with the fractional softening values obtained through Equation (1), from the hardness measurement results according to the annealing time. Both the initial hardness (*H*_0_) and the hardness after complete REX (*H_fullrex_*) have the same value in the same reduction condition, when determining the softening fraction (*X_H_*) according to the time under the conditions used in this study. Therefore, the hardness of (*H_anneal_*) served as the final factor determining the value of the softening fraction, and an increase in the softening fraction result in a decrease in hardness. In this graph, the softening fraction was increased under conditions where the annealing temperature or the reduction ratio increased. In particular, when annealing was performed at 723 K for both reduction rates, the softening fraction initially increased, and then gradually increased or partially decreased thereafter, while showing a larger value than the opposite condition.

**Figure 10:**
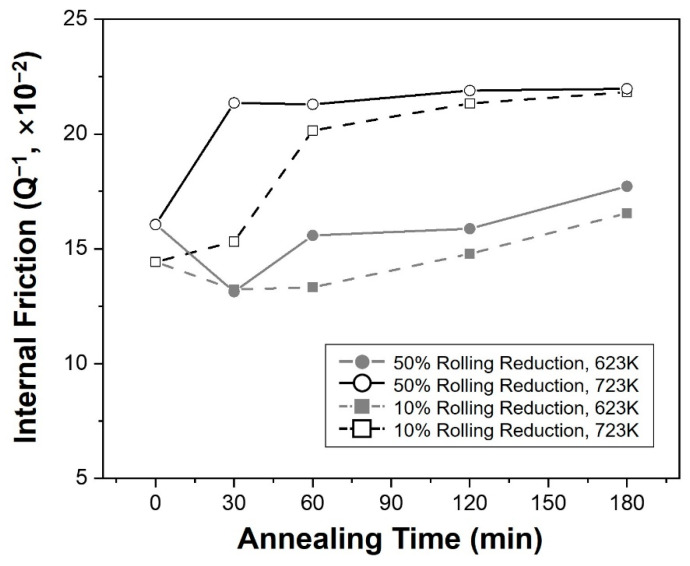
Graph showing the internal friction of specimens before and after the annealing process along with the annealing time.

Figure 10 shows the variation of the internal friction coefficient measured from each specimen along with the annealing time. As a result, different behaviors were observed depending on the annealing temperature. The internal friction maintained almost the same after greatly increasing in the initial stage for the specimens annealed at 723 K, while the specimens at 623 K decreased in the initial stage of annealing and increased progressively.

## 4. Discussion

### 4.1. Deformation Mechanism

AZ31 was deformed at 673 K until each rolling reduction. Barnett et al. has investigated the degree of activation of each slip system in Mg alloy, and reported that the non-basal slip systems became activated over the 688 K and their critical resolved shearing stresses (CRSS) were almost same with the basal system [18]. However, because the rolling was conducted at 673 K, the occurrence of the same effect was hard to estimate in this study. Therefore, the basal <a> slip system seemed to be mostly activated, and twinning systems, especially tensile twinning would be activated additionally to accompany the deformation [19].

From the results, more twins were observed in the microstructure when the reduction ratio increased, and as shown in Section 3.2, coarse-grain textures had an orientation tilted from the (0001). The development of tilted texture in AZ31 has been reported in the compression results of AZ31 [17]. Moreover, the grains having lower size than average showed the strong [11$\bar{2}$0] component. These results well supported the assumption.

In addition, due to annealing, REX was generated in the form of newly occurring recrystallized grains in twinning. The higher the fraction of twins, temperature, and time of annealing, the larger the REX behavior observed. These implied that the tensile twins provided the main site for REX generation during annealing.

### 4.2. Static Recrystallization

Generally, static recrystallization is classified into three stages according to the change in grain size [20]. First, in the initial stage of annealing, an incubation period in which the grain size barely changes is followed by a period of rapid grain change in which the grain size changes significantly. Subsequently, the grain size gradually increased throughout the crystal grain growth stage until reaching the final stage.

Hot rolling formed many twins inside the microstructure of AZ31, and more twins were formed as the reduction ratio increased. Conversely, the annealing treatment caused static REX in these twins. Therefore, the presence of twins and high temperatures acted as a driving force for REX. In addition, the increase in annealing time contributed to the increase in REX by supplying a continuous driving force as REX progressed. Furthermore, fine crystal grains were formed from the inside of the twin by REX; the size of the crystal grains comprising existing twin and parent crystal grains demonstrated a considerably coarse grain diameter, while the recrystallized grains showed a lower grain diameter value. Therefore, from the observation results in Section 3.2, crystal grains larger than those with average diameter were regarded as crystal grains owing to the existing deformation, while crystal grains below the standard were regarded as recrystallized grains.

A high strength toward the [11$\bar{2}$0] orientation was observed because the orientation of the fine crystal grains in the observed texture was mostly attributed to the tensile twins after rolling. Conversely, the orientation of such grains was eliminated, and the grains were realigned towards the basal during REX annealing. Additionally, the orientation change during REX was mainly because of the nucleation of the discontinuous type [17,21].

### 4.3. Damping Capacity

**Figure 11:**
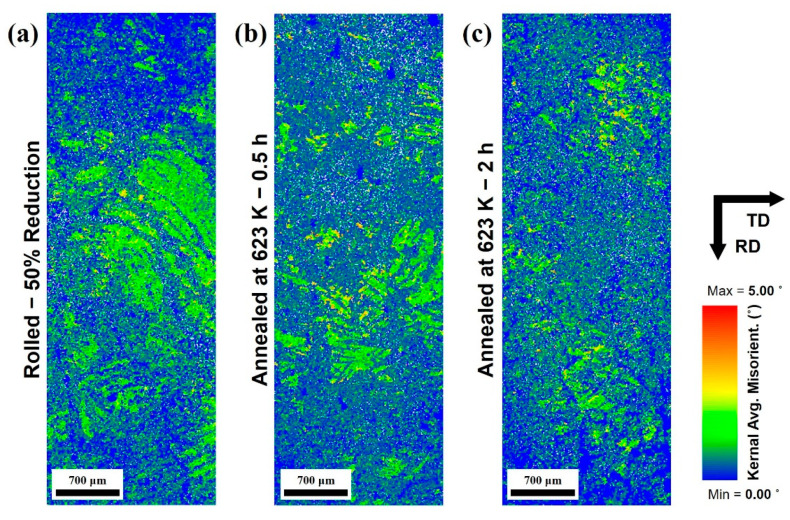
Kernal average misorientation (KAM) maps of the (**a**) rolled with 50% reduction and annealed at 623 K for (**b**) 0.5 and (**c**) 2 h.

Figure 11 shows the kernal average misorientation (KAM) map of the full-scale microstructures shown in Figure 5. The microstructure of specimens were used, which are rolled with 50% reduction and the ones annealed at 623 K for 0.5 and 2 h. In addition, the misorientation in the range from 0 to 5° was shown as a color point in the KAM image. Because the misorientation shows the local deformation and the major deformation mechanism in this study was twinning and dislocation, the local area having especially high misorientation value to be regarded as the area as the concentrated dislocation density area.

The highest value of dislocation densities is observed in the twinning grains as shown in the Figure 11a, which is rolled at 50% reduction. On the other hand, as shown in Figure 11b, the concentrated misorientation zones were dissipated in the grains after the annealing was conducted. In addition, when the annealing time was increased, this behavior became stronger while the recrystallized grains increased in size at the same time.

As mentioned in the introduction, the damping ability of magnesium alloys is generally well known as a dislocation-type damping mechanism, and this characteristic appears when mechanisms that hinder the movement of dislocations act as pinning points [4,6]. They can be distinguished into two categories; weak pinning points including solute atoms and vacancies, and strong pinning points such as grain or phase boundaries. In this study, considering that the same alloy was used and that temperature was also included under the same conditions, the effect of the strong pinning point became an important factor. In addition, the distance between grain boundaries and the dislocation density inside the twins would determine the internal friction, because the formation of twins by rolling and REX through subsequent annealing were the main factors that changed the microstructures of the alloy. Furthermore, the dislocation densities decreased correspondingly, because of the recovery during the annealing process in the results of this study. On the contrary, more fine crystal grains were generated during the REX process, which is critical for further reducing the distance between strong pinning points. In the measurement results of the internal friction, more twins appeared in the specimens immediately after rolling as the reduction ratio increased from 10% to 50%, and this increase strengthened internal friction. However, during the annealing process, different behaviors were observed depending on the annealing temperature. When the annealing was conducted at 723 K, the crystal grains were refined to approximately half due to static REX inside the twin, and the internal friction continuously increased gradually. This increased the distance between grain boundaries as it gradually grew after recovery and recrystallization occurred at the initial stage. Moreover, in the Vickers hardness test, which is affected by the dislocation density, the softening fraction increased considerably through this process, indicating that the dislocation density was highly resolved and had little effect.

On the other hand, annealing at a lower temperature relatively slowed REX of the twins, and the crystal grains were gradually refined throughout the entire process at 623 K. Consequently, the internal friction decreased once in the initial stage of annealing and then increased again under both reduction conditions. Dislocation densities concentrated inside the twins disappeared to form new grains, but due to the shortage of driving force, these dislocation densities did not completely disappear, and they seemed to contribute to the reduction of the friction coefficient. In the subsequent increase, the fraction of recrystallized grains gradually increased as the dislocation density was resolved, and it seemed to show the same increase as they underwent the grain growth.

The effects of rolling and annealing treatment of magnesium alloys on the vibration-damping ability were discussed in terms of twin and static REX development. Furthermore, it was confirmed that various processing methods for Mg alloys can contribute to an increase or decrease in the internal friction coefficient. There is a correlation between texture and vibration damping ability, considering that a texture with a specific orientation is formed through the control of various factors during the processing of magnesium alloys. However, further research is required to confirm these.

## 5. Conclusions

Rolling with two different reductions and annealing under different time and temperature conditions were conducted to investigate the effect of rolling reduction and annealing condition on the damping capacity of AZ31 magnesium alloy. Their damping capacity was studied through the results of the microstructures and textures using SEM-EBSD. The major results are as follows;

1. A tensile twin was developed in hot rolled AZ31 magnesium alloy by high-temperature rolling, and its fraction increased along with an increase in rolling reduction.
2. The tensile twin disappeared and equiaxial grains were formed by recrystallization, this behavior was more obvious when the annealing time and temperature were increased.
3. A tilted texture from basal and tensile twin texture were formed through the rolling. However, they were realigned toward the (0001) orientation by recrystallization during the annealing process.
4. The higher fraction of tensile twinning owing to the increase in rolling reduction or the higher annealing temperature and time provided a higher driving force for recrystallization. Thus, the magnitude of recrystallization affected the damping capacity.
5. Dislocation densities affected by the twinning and annealing was the major factor controlling the damping properties. The development of twinning increased the internal friction by increasing the dislocation density. Annealing temperature varied the average grain size by controlling the magnitude of static recrystallization. The distances between grain boundary changed the internal friction values.

## Figures and Tables

**Table 1:** Chemical composition of AZ31 alloy used in this study.

Element	Mg	Al	Zn	Mn	Si
Component	Bal.	3.20	0.79	0.19	0.05
					(wt%.)

## Data Availability

The datasets generated during and/or analyzed during the current study are available from the corresponding author on reasonable request.
